# Epidemiology of biliary tract-associated bloodstream infections and adequacy of empiric therapy: an Australian population-based study

**DOI:** 10.1007/s10096-024-04894-9

**Published:** 2024-07-10

**Authors:** Ian Gassiep, Felicity Edwards, Kevin B. Laupland

**Affiliations:** 1 Faculty of Medicine, UQ Centre for Clinical Research, The University of Queensland, Brisbane, QLD Australia; 2 Department of Infectious Diseases, Mater Hospital Brisbane, Brisbane, QLD Australia; 3 Pathology Queensland, Royal Brisbane & Women’s Hospital, Brisbane, QLD Australia; 4 Department of Intensive Care Services, Royal Brisbane and Women’s Hospital, Level 3 Ned Hanlon Building, Brisbane, QLD Australia; 5 Queensland University of Technology (QUT), Brisbane, QLD Australia

**Keywords:** Biliary tract infection, Bloodstream infection, Cholecystitis, Cholangitis, Empiric therapy

## Abstract

**Purpose:**

Although the biliary tract is a common source of invasive infections, the epidemiology of cholangitis- and cholecystitis-associated bloodstream infection (BSI) is not well defined. The objective of this study was to determine the incidence, clinical determinants, microbiology of biliary tract-associated BSI, and predicted adequacy of common empiric therapy regimens.

**Methods:**

All biliary tract-associated BSI in Queensland during 2000–2019 were identified using state-wide data sources. Predicted adequacy of empiric antimicrobial therapy was determined according to microbiological susceptibility data.

**Results:**

There were 3,698 episodes of biliary tract-associated BSI occurred in 3,433 patients of which 2,147 (58.1%) episodes were due to cholangitis and 1,551 (41.9%) cholecystitis, for age- and sex-standardized incidence rates of 2.7, and 2.0 per 100,000 population, respectively. An increasing incidence of biliary tract-associated BSI was observed over the study that was attributable to an increase in cholangitis cases. There was a significant increased risk for biliary tract-associated BSI observed with advancing age and male sex. Patients with cholangitis were older, more likely to have healthcare associated infection, and have more comorbidities most notably liver disease and malignancies as compared to patients with cholecystitis. The distribution of infecting pathogens was significantly different with polymicrobial aetiologies more commonly observed with cholangitis (18.4% vs. 10.5%; *p* < 0.001). The combination of ampicillin/gentamicin/metronidazole was predicted to have the overall highest adequacy (96.1%), whereas amoxicillin/clavulanate had the lowest (77.0%). Amoxicillin/clavulanate (75.2% vs. 79.4%, p:0.03) and ceftriaxone/metronidazole (83.4% vs. 89.6%; *p* < 0.001) showed significantly inferior predicted adequacy for cholangitis as compared to cholecystitis.

**Conclusions:**

Bloodstream infections related to cholecystitis and cholangitis exhibit different epidemiology, microbiology, and requirements for empiric therapy.

**Supplementary Information:**

The online version contains supplementary material available at 10.1007/s10096-024-04894-9.

## Introduction

In Australia, gallbladder pathology is common. A recent study revealed that there were over 1 million hospitalisations due to symptomatic gallbladder-associated disease and almost 800,000 cholecystectomies performed from 2004 to 2019 [1]. Biliary tract infections (BTI) are a common cause of gladder-associated hospitalisation and can be categorised as cholecystitis or cholangitis [2, 3].

Acute cholecystitis, inflammation of the gallbladder, may be defined as a combination of local signs such as positive Murphy’s sign, systemic features including fever or elevated white cell count, and imaging findings characteristic of cholecystitis [3]. Approximately 90% of acute cholecystitis episodes are related to gallstones [4]. Cholangitis, inflammation of the bile duct system, may occur as a result of biliary obstruction, often due to stones within the biliary duct or due to malignancy. Acute cholangitis may be defined as systemic inflammation including fever and laboratory evidence of inflammation, cholestasis including jaundice or abnormal liver function tests, and imaging demonstrating biliary dilatation or aetiological evidence such as stricture or stone [2].

BTIs are commonly associated with bloodstream infections (BSI) [5, 6]. Multiple studies have demonstrated the most common causative pathogens include *Escherichia coli*, *Klebsiella* sp., and *Enterococcus* sp. [7–9]. Rarely, organisms such as *Pseudomonas aeruginosa*, *Enterobacter cloacae*, and anaerobic bacteria are also associated with bacteraemia [7–10]. Management of BTI involves antimicrobials, supportive therapies, and source control. Definitive therapy involves cholecystectomy for cholecystitis and endoscopic retrograde cholangiopancreatography (ECRP) for cholangitis. In some cases percutaneous drainage may be performed [11, 12].

Australia has a national therapeutic guideline that provides empiric antimicrobial therapy advice for common presentations including both cholecystitis and cholangitis [13]. There are limited data regarding the causative pathogens of bloodstream infection in patients with BTI in Australia. Therefore, the current national guidelines are based on international microbiological data, expert opinion, and local antimicrobial resistance rates [14]. The authors aimed to provide epidemiological data relating to BTI-associated bloodstream infections in a large Australian population; identify the causative pathogens for these clinical syndromes; and determine if the current Australian guidelines for empiric antimicrobial management are appropriate.

## Methods

A population-level retrospective cohort study including all patients admitted to any Queensland public hospital was performed. The study occurred between 1 January 2000 and 31 December 2019, and included all patients with cholecystitis or cholangitis associated with a community-onset bloodstream infection. Patients were identified through the state-wide electronic pathology system (AUSLAB). Bloodstream infection was defined by the isolation of a bacterium or yeast from a blood culture from a patient. Repeat isolations within 30-days were considered to represent the same episode. Polymicrobial bacteraemia was defined as ≥ 2 pathogens drawn within 48 h. Community-onset bloodstream infection was defined as a positive blood culture drawn prior to or within 48-hours of hospital admission and was further classified as healthcare-associated if associated with prior significant healthcare exposure as defined by Friedman et al. [15–17]. Patients were excluded if only a single positive blood culture with a common skin contaminant (i.e. coagulase negative Staphylococci, *Bacillus sp.*, *Corynebacterium sp*., *Cutibacterium acnes*, *Propionibacterium sp*., or viridans group Streptococci (excluding *Streptococcus anginosus* group)) was identified [18].

Basic demographic, admissions information, and diagnostic and comorbidity information was obtained by linkage to the state-wide hospital admissions database. We identified patients with biliary tract-associated infections from within the overall population of patients with bloodstream infection using ICD10 AM discharge codes “K8040” or “K8000” or “K8001” or “K801” or “K8010” or “K8011” or “K803” or “K8030” or “K839” or “K804” or “K800” or “K8041” or “K810” or “K811” or “K818” or “K819” or “K830” or “K831” or “K832” or “K8031”. Data relating to diagnosis of acalculous and calculous cholecystitis was not available. Therefore, these diagnoses have been combined as “cholecystitis” hereafter.

The empiric antimicrobial regimens assessed in this study were selected based on a combination of national and international guidelines [13, 19]. An adjudication of predicted adequate empiric therapy in relation to the microbiological data was made for each case based on consensus of two infectious diseases consultants. Where organisms were demonstrated to be susceptible to one or more of the antibiotic regimens this was deemed to represent predicted adequacy of treatment, with the exception of ciprofloxacin for *Enterococcus sp.* or *Staphylococcus aureus* for which this was deemed inadequate. For isolates where testing was not available, intrinsic resistance (i.e. cephalosporin resistance in enterococci) or typical patterns of susceptibility (i.e. >95% metronidazole susceptibility for most anaerobes) was used to make a decision. For polymicrobial infections, regimens were deemed to be inadequate if any of the isolates were non-susceptible. Susceptibility results were interpreted using The European Committee on Antimicrobial Susceptibility (EUCAST), version 13. Due to changes in interpretation and reporting over time, non-resistant isolates were classified as susceptible (i.e. combining S – susceptible and I – susceptible, increased exposure). Identification of extended spectrum β-lactamase (ESBL) activity was performed by the combination disc method as outlined by EUCAST [20].

The human research ethics committee at the Royal Brisbane and Women’s Hospital approved the study and granted a waiver of individual informed consent (LNR/2020/QRBW/62,494).

### Statistical analysis

Data was analysed using Stata 17 (StataCorp, College Station, USA). The unit of analysis was incident BTI-associated BSI episodes, and were reported as age- and sex-standardised (2019 Queensland population) annual rates per 100,000 population. Denominator data were obtained from Queensland Health. The total annual number of sets of bloods cultures performed was obtained from Pathology Queensland. Incidence rate ratios (IRR) with exact 95% confidence intervals (CI) were calculated for group comparison. Categorical data were analysed using the Chi-square test. Skewed continuous variable were reported as medians with interquartile ranges (IQR) and compared using Wilcoxon-Mann-Whitney tests. P values < 0.05 were deemed to be statistically significant.

## Results

There were 3,698 episodes of biliary tract-associated BSI among 3,433 Queensland residents, with 210 (6.1%), 32 (0.9%), 13 (0.4%), six (0.2%), three (0.1%), and one (< 0.1%) having two, three, four, five, six, or seven incident episodes, respectively. The majority (2,435; 65.9%) were classified as community-associated and 1,263 (34.2%) were healthcare-associated. Overall, 2,147 (58.1%) were diagnosed with cholangitis and 1,551 (41.9%) with cholecystitis.

**Fig. 1:**
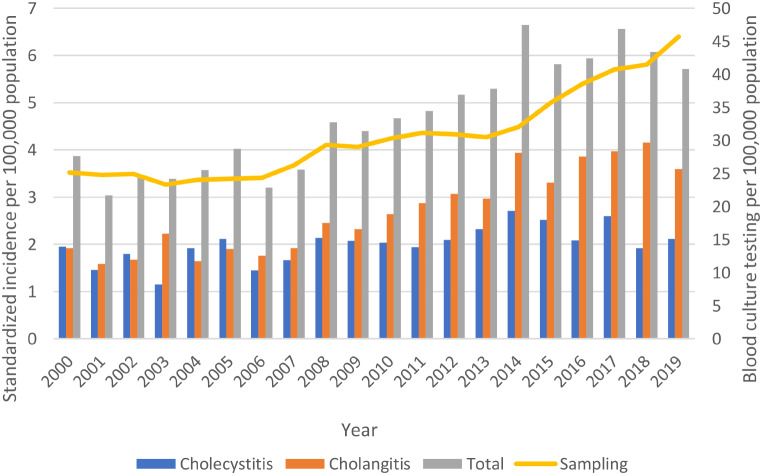
Age-and sex-standardized incidence of biliary tract-associated bloodstream infections in Queensland, Australia, 2000–2019

The age- and sex-standardized incidence of biliary tract-associated BSI was 4.7 per 100,000 population, and this was 2.0 and 2.7 per 100,000 annually for cholecystitis and cholangitis, respectively. During the twenty-year study there was an increasing incidence of biliary tract-associated BSI that was attributable to an increase in cholangitis as shown in Fig. 1. No monthly or seasonal variation trends in occurrence were observed.

**Fig. 2:**
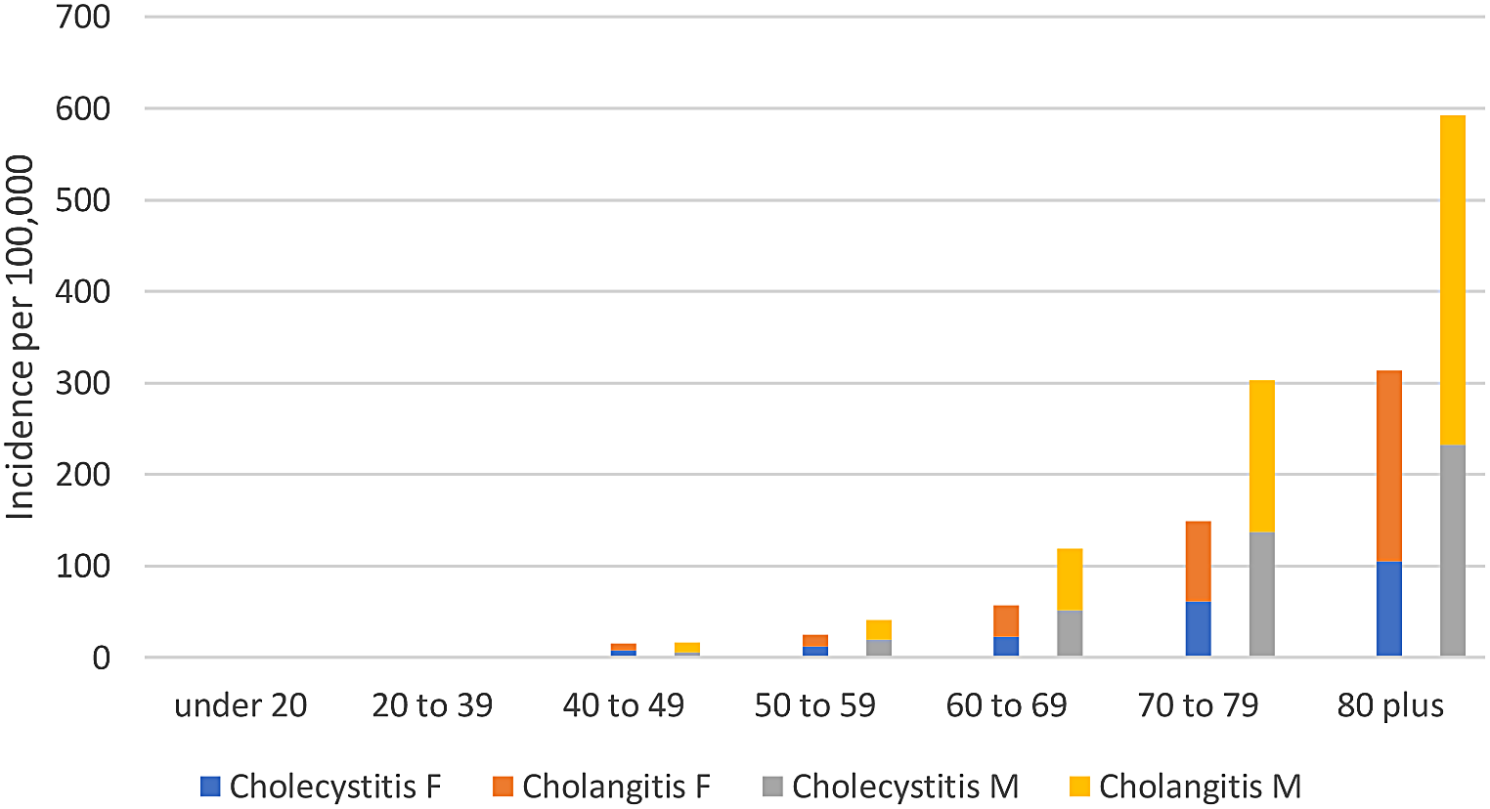
Age- and sex-related incidence of biliary tract associated bloodstream infections, Queensland, Australia, 2000–2019

There was a significant increased risk for BTI-associated BSI observed with advancing age and male sex as shown in Fig. 2. BTI-associated BSI was rare in those aged less than 40 (45; 1.2%) years of age. All 13 cases occurring among those aged less than 20 years were due to cholangitis of which nine occurred in those aged less than one year all of whom had underlying comorbidities. An overall higher incidence was observed in males as compared to females (5.2 vs. 3.3 per 100,000; IRR 1.6; 95% CI, 1.47–1.68), with the excess risk in males related to the oldest age groups (Fig. 2). Similar risks related to age and sex were observed with both cholecystitis and cholangitis as shown in Fig. 2.

Patients with cholangitis were older, more likely to have healthcare-associated infections, and had more co-morbid illnesses than patients with cholecystitis as shown in Table 1. Although the number with mild to moderate comorbidity were similar, patients with cholecystitis were more likely to have a zero Charlson comorbidity index score and less likely to have severe (i.e. 5 or more) scores as shown in Table 1. The differences in comorbidities were driven predominantly by significantly higher rates of cancer and liver disease among patients with cholangitis (Table 1).

**Table 1:** Clinical features of biliary tract-associated bloodstream infection

Factor	Cholecystitis (*n* = 1,551)	Cholangitis (*n* = 2,147)	*P*-value
Median age (interquartile range)	73.4 (62.9–80.9)	75.4 (65.6–83.1)	< 0.001
Male sex (%)	973 (62.5)	1,287 (59.9)	0.13
Infection onset (%)			< 0.001
Healthcare-associated	425 (27.4%)	838 (39.0%)	
Community-associated	1126 (72.6%)	1309 (61.0%)	
Median Charlson comorbidity index (interquartile range)	1 (0–2)	2 (0–4)	< 0.001
Charlson comorbidity index scores			< 0.001
0	666 (42.9)	751 (35.0)	
2–3	499 (32.2)	641 (29.9)	
3–4	217 (14.0)	331 (15.4)	
≥ 5	169 (10.9)	424 (19.8)	
Myocardial infarction	137 (8.8)	178 (8.3)	0.59
Congestive heart failure	201 (13.0)	239 (11.1)	0.10
Peripheral vascular disease	71 (4.6)	88 (4.1)	0.51
Dementia	66 (4.3)	104 (4.8)	0.43
Pulmonary disease	170 (11.0)	193 (9.0)	0.050
Rheumatic disease	17 (1.1)	24 (1.1)	1.0
Peptic ulcer disease	31 (2.0)	31 (1.4)	0.20
Plegia	47 (3.0)	43 (2.0)	0.051
Renal disease	195 (12.6)	250 (11.6)	0.40
Human immunodeficiency virus	1 (0.1)	1 (0.1)	1.0
Malignancy (%)			< 0.001
None	1445 (93.2)	1681 (78.3)	
No metastasis	74 (4.8)	233 (10.9)	
Metastasis	32 (2.1)	233 (10.9)	
Diabetes	421 (17.2)	544 (25.3)	0.23
Liver disease (%)			< 0.001
None	1434 (92.5)	1900 (88.5)	
Mild	79 (5.1)	137 (6.4)	
Severe	38 (2.5)	110 (5.1)	

### Microbiology

A total of 4,404 incident isolates were obtained from 3698 episodes of bloodstream infection. The microbiology of all isolates overall is displayed in Supplementary Table 1. Monomicrobial aerobic Gram negatives accounted for 2, 833/3698 (77%) of infections. Polymicrobial infections accounted for 559/3,698 (15%) episodes. Cholangitis was associated with nearly twice the risk for polymicrobial infections as compared to cholecystitis (396/2,147; 18.4% vs. 163/1,551; 10.5%; *p* < 0.001). ESBL-producing organisms were only identified in 1.6% of patients (52/2,283 *E. coli* and 7/682 *K. pneumoniae*), and 95% (56/59) occurred after 2010. Organisms considered to have moderate to high risk of clinically significant inducible AmpC enzyme production including *Hafnia alvei*, *Enterobacter cloacae* complex, *Citrobacter freundii* complex, *and Klebsiella aerogenes* (HECK organisms) accounted for 5.5% (244/4,404) of isolates in 6.5% of patients (241/3,698) patients. Vancomycin-resistant Enterococci (VRE) were rare and accounted for 0.1% (5/4,404) of organisms identified. Although most isolates were Enterobacterales, there was a significant difference in distribution of organisms (*p* < 0.001) causing biliary tract-associated bloodstream infection among cholecystitis and cholangitis cases (Table 2).

**Table 2:** Organisms causing biliary tract-associated bloodstream infection

Organism	Cholecystitis (*n* = 1551)	Cholangitis (*n* = 2147)
*Escherichia coli*	823 (53.1%)	1055 (49.1%)
*Klebsiella* species	242 (15.6%)	355 (16.5%)
Other Enterobacterales	98 (6.3%)	147 (6.9%)
Anaerobes	59 (3.8%)	20 (0.9%)
*Enterococcus* species	38 (2.5%)	58 (2.7%)
*Staphylococcus aureus*	33 (2.1%)	11 (0.5%)
*Streptococcus* anginosus group	31 (2.0)	17 (0.8)
Other Gram negatives	23 (1.5%)	39 (1.8%)
Other streptococci	17 (1.1)	7 (0.3)
*Pseudomonas species*	12 (0.8%)	39 (1.8%)
Other Gram positives	11 (0.7)	3 (0.1)
*Candida rugosa*	1 (0.1%)	0
Polymicrobial	163 (10.5)	396 (18.4)

### Predicted adequacy of common empiric treatment regimens

The predicted adequacies of empiric treatment regimens commonly recommended by guidelines for 3,698 biliary tract-associated bloodstream infection episodes are shown in Table 3. The combination of ampicillin (AMP)/gentamicin (GEN)/metronidazole (MET) had the overall highest adequacy (3,552; 96.1%); whereas amoxicillin/clavulanate (AMC) had the lowest (2,846; 77.0%). There was no statistically significant difference in pathogens and therefore adequacy of therapy between community-associated and healthcare-associated infections. Notably the adequacy of empiric therapies were significantly different between cholecystitis and cholangitis with ceftriaxone (CTR)/MET and AMC demonstrating lower adequacy for cholangitis (Table 3). Overall, AMC demonstrated the highest rate of inadequate treatment (852/3,698, 23%), with the majority of these occurring in monomicrobial infections (498/852, 58%). A primary contributing factor to AMC inadequacy was *E. coli* resistance. 51% (254/498) of *E. coli* isolates were resistant to AMC. In vitro susceptibility of the HECK organisms was demonstrated for 78% (190/244) PIT, 75% CTR (182/244), and 8% AMC (20/244), respectively.

**Table 3:** Adequacy of commonly recommended empiric regimens for biliary tract-associated bloodstream infections

Regimen	Cholecystitis (*n* = 1551)	Cholangitis (*n* = 2147)	*p*-value
AMP + GEN + MET	1485 (95.7%)	2067 (96.3%)	0.44
CTR + MET	1389 (89.6%)	1791 (83.4%)	< 0.001
PIT	1463 (94.3%)	1994 (92.9%)	0.080
AMC	1231 (79.4%)	1615 (75.2%)	0.003
CIP + MET	1427 (92.0%)	1966 (95.6)	0.67

Antimicrobial abbreviations: AMP: ampicillin; GEN: gentamicin; MET: metronidazole; CTR: ceftriaxone; PIT: piperacillin-tazobactam; AMC: amoxicillin-clavulanate; CIP: ciprofloxacin

## Discussion

This study reveals an increasing incidence of biliary tract infection-associated bloodstream infections in a large Australian population. Age was found to be a significant risk factor in our cohort, with the majority of infections occurring in those over 70 years. Notably, Queensland has an ageing population, with those aged over 65 years increasing by 3.6% from 2011 to 2021 [21]. Given the growth of this at-risk population, the incidence of BTI-associated bloodstream infection is likely to continue to increase.

We report a higher incidence of bloodstream infection associated cholangitis as compared to cholecystitis. While this is similar to previous international data, the proportion of cholangitis cases (58%) in our study is lower than the 77–82% reported elsewhere [7, 22]. Similarly, only 11% of patients in our cohort had an underlying malignancy as compared to 25–41%, which may provide a partial explanation for the aforementioned difference in cholangitis incidence [7, 8, 22].

As expected, monomicrobial Gram negative infection was the most common microbiological diagnosis for BTI-associated BSI. Consistent with previous publications, polymicrobial infections were common. Enterococci, which are reported to cause 8–20% of infections were less frequent in our cohort with involvement of 2.5–2.7% of monomicrobial episodes (Table 2) and 4.5–8.1% of overall isolates (Supplementary Table 1) [7–9, 22]. Additionally, *Pseudomonas sp*. only accounted for approximately 1% in cholecystitis and 2% of infections in cholangitis patients which is lower than observed in previously published results [9, 19]. The reason for discrepancies in pathogen incidence are unclear, but may be related to regional differences. Alternatively, the comparative analysis may be affected by sample size. Our cohort consists of over 3,500 episodes of BTI-associated bloodstream infection. Whereas, the sample sizes of international studies ranged from 75 to 568 patients [9, 23].

The current Australian guideline recommendation for management of BTIs is differentiated according to aetiology [13]. Empiric antimicrobial therapy for calculous cholecystitis suggests intravenous (IV) gentamicin and ampicillin; amoxicillin-clavulanate; or ceftriaxone. The guideline states that acute acalculous cholecystitis is uncommon and that pathogens associated with this presentation include Enterobacterales, streptococci, *Pseudomonas aeruginosa*, and anaerobes. Therefore, empiric therapy for this condition includes ampicillin, gentamicin, and metronidazole or piperacillin-tazobactam monotherapy. Finally, empiric therapy for acute cholangitis includes ampicillin and gentamicin; ceftriaxone; or piperacillin-tazobactam. In the present study cholecystitis is not classified by aetiology. However, taken together, the combination of ampicillin, gentamicin, and metronidazole was the most likely to provide adequate antimicrobial cover. The current guideline recommendation to not include specific anaerobic cover for cholangitis appears reasonable.

Previous guidelines have suggested that where a common community-acquired pathogen demonstrates 10–20% resistance to a given antimicrobial, then this agent should be avoided [19, 24]. While piperacillin-tazobactam and amoxicillin-clavulanate have similar spectra of activity, these agents do not have the same in vitro activity [25]. The data presented in this study suggest a potential risk of antimicrobial mismatch for 1 in 5 patients treated with amoxicillin-clavulanate for cholecystitis. Additionally, the role of ceftriaxone and metronidazole in the setting of cholangitis could be considered questionable. The need for an anti-pseudomonal agent for either presentation may not be justified given the low incidence in this setting.

The incidence of multi-resistant organisms in our cohort is low. The rate of ESBL-producing *E.coli* (2.2%) is lower in BTI-associated bloodstream infection patients compared to previous population-based BSI data (4.2%) from Queensland [26]. This is likely attributable to the fact that this study reported on a substantial number of urinary tract-associated BSIs. However, similar to our results, the previous study demonstrated an increase in incidence of ESBL-producing isolates from 2010 onwards [26]. As the rate of increase was reported as 25% per year, it would be pertinent to continue to review and potentially adjust local empiric therapy guidelines within the next 10 years.

The current Infectious Diseases Society of America (IDSA) management guideline recommends against the use of CTR or PIT for invasive or serious infections caused by organisms at moderate or high risk of clinically significant AmpC production [27]. Currently, the incidence of these organisms is < 10% in our cohort. Importantly, there is limited evidence regarding patient outcomes in the setting of empiric therapy with these agents followed by switch to an appropriate alternative agent. Therefore, even in the event of a future increase in incidence of these organisms, an empiric therapy adjustment may not be warranted.

There are several limitations in this study. Due to the retrospective nature of the analysis all clinical data could not be confirmed. Data relating to cause or chronicity of cholangitis or cholecystitis was not assessable. Additionally, patient treatment information was not available, and therefore treatment outcomes data could not be assessed. The dataset for this study was created from positive blood culture results. Therefore, a denominator for total number of patients with BTI-associated BSI could not be determined. The diagnoses in this analysis were generated from discharge codes, and therefore errors in coding may have occurred. Australia is in a fortunate position with regards to the low prevalence of multidrug resistant Enterobacterales in the community. Consequently, the data presented in this study are not generalizable to many regions. Notwithstanding these limitations, this study provides substantial epidemiological and microbiological data related to BTI-associated BSI.

## Conclusion

The incidence of biliary tract-associated bloodstream infection is increasing in Queensland, Australia. The relative rates of pathogens associated with these infections differ from previously reported international data. Our findings suggest that empiric amoxicillin/clavulanate therapy may not be appropriate in this setting. Prospective clinical studies are required to define clinical outcomes and aid in refining future antimicrobial prescribing guidelines.

### Electronic supplementary material

Below is the link to the electronic supplementary material.

Supplementary Material 1

## Data Availability

Data cannot be shared publicly due to institutional ethics, privacy, and confidentiality regulations. Data release for the purposes of research under Section. 280 of the Public Health Act 2005 requires application to the Director General (PHA@health.qld.gov.au).

## References

[1] Mollah T, Christie H, Chia M, Modak P, Joshi K, Soni T, Qin KR (2022) Gallbladder-associated hospital admission and cholecystectomy rates across Australia and Aotearoa New Zealand (2004–2019): are we over-intervening? Ann Hepatobiliary Pancreat Surg 26(4):339–346 35383131 10.14701/ahbps.22-007 PMC9721247

[2] Kiriyama S, Kozaka K, Takada T, Strasberg SM, Pitt HA, Gabata T, Hata J, Liau K-H, Miura F, Horiguchi A, Liu K-H, Su C-H, Wada K, Jagannath P, Itoi T, Gouma DJ, Mori Y, Mukai S, Giménez ME, Huang WS-W, Kim M-H, Okamoto K, Belli G, Dervenis C, Chan ACW, Lau WY, Endo I, Gomi H, Yoshida M, Mayumi T, Baron TH, de Santibañes E, Teoh AYB, Hwang T-L, Ker C-G, Chen M-F, Han H-S, Yoon Y-S, Choi I-S, Yoon D-S, Higuchi R, Kitano S, Inomata M, Deziel DJ, Jonas E, Hirata K, Sumiyama Y, Inui K, Yamamoto M (2018) Tokyo guidelines 2018: diagnostic criteria and severity grading of acute cholangitis (with videos). J Hepato-Biliary-Pancreat Sci 25(1):17–30 10.1002/jhbp.512 29032610

[3] Yokoe M, Hata J, Takada T, Strasberg SM, Asbun HJ, Wakabayashi G, Kozaka K, Endo I, Deziel DJ, Miura F, Okamoto K, Hwang T-L, Huang WS-W, Ker C-G, Chen M-F, Han H-S, Yoon Y-S, Choi I-S, Yoon D-S, Noguchi Y, Shikata S, Ukai T, Higuchi R, Gabata T, Mori Y, Iwashita Y, Hibi T, Jagannath P, Jonas E, Liau K-H, Dervenis C, Gouma DJ, Cherqui D, Belli G, Garden OJ, Giménez ME, de Santibañes E, Suzuki K, Umezawa A, Supe AN, Pitt HA, Singh H, Chan ACW, Lau WY, Teoh AYB, Honda G, Sugioka A, Asai K, Gomi H, Itoi T, Kiriyama S, Yoshida M, Mayumi T, Matsumura N, Tokumura H, Kitano S, Hirata K, Inui K, Sumiyama Y, Yamamoto M (2018) Tokyo guidelines 2018: diagnostic criteria and severity grading of acute cholecystitis (with videos). J Hepato-Biliary-Pancreat Sci 25(1):41–54 10.1002/jhbp.515 29032636

[4] Strasberg SM (2008) Acute Calculous Cholecystitis. N Engl J Med 358(26):2804–2811 18579815 10.1056/NEJMcp0800929

[5] Melzer M, Toner R, Lacey S, Bettany E, Rait G (2007) Biliary tract infection and bacteraemia: presentation, structural abnormalities, causative organisms and clinical outcomes. Postgrad Med J 83(986):773–776 18057178 10.1136/pgmj.2007.064683 PMC2750926

[6] García-Rodríguez JF, Mariño-Callejo A (2023) The factors associated with the trend in incidence of Bacteraemia and associated mortality over 30 years. BMC Infect Dis 23(1):69 36737678 10.1186/s12879-023-08018-0 PMC9897612

[7] Ortega M, Marco F, Soriano A, Almela M, Martínez JA, López J, Pitart C, Mensa J (2012) Epidemiology and prognostic determinants of bacteraemic biliary tract infection. J Antimicrob Chemother 67(6):1508–1513 22408140 10.1093/jac/dks062

[8] Tan M, Jensen TG, Nielsen SL, Schaffalitzky de Muckadell OB, Laursen SB (2021) Analysis of patterns of bacteremia and 30-day mortality in patients with acute cholangitis over a 25-year period. Scand J Gastroenterol 56(5):578–584 33764841 10.1080/00365521.2021.1902558

[9] Jeong HT, Song JE, Kim HG, Han J (2022) Changing patterns of causative pathogens over Time and efficacy of empirical antibiotic therapies in Acute Cholangitis with Bacteremia. Gut Liver 16(6):985–994 35321958 10.5009/gnl210474 PMC9668498

[10] Otani T, Ichiba T, Seo K, Naito H (2022) Blood cultures should be collected for acute cholangitis regardless of severity. J Infect Chemother 28(2):181–186 34635451 10.1016/j.jiac.2021.10.004

[11] Miura F, Okamoto K, Takada T, Strasberg SM, Asbun HJ, Pitt HA, Gomi H, Solomkin JS, Schlossberg D, Han HS, Kim MH, Hwang TL, Chen MF, Huang WS, Kiriyama S, Itoi T, Garden OJ, Liau KH, Horiguchi A, Liu KH, Su CH, Gouma DJ, Belli G, Dervenis C, Jagannath P, Chan ACW, Lau WY, Endo I, Suzuki K, Yoon YS, de Santibañes E, Giménez ME, Jonas E, Singh H, Honda G, Asai K, Mori Y, Wada K, Higuchi R, Watanabe M, Rikiyama T, Sata N, Kano N, Umezawa A, Mukai S, Tokumura H, Hata J, Kozaka K, Iwashita Y, Hibi T, Yokoe M, Kimura T, Kitano S, Inomata M, Hirata K, Sumiyama Y, Inui K, Yamamoto M (2018) Tokyo guidelines 2018: initial management of acute biliary infection and flowchart for acute cholangitis. J Hepatobiliary Pancreat Sci 25(1):31–40 28941329 10.1002/jhbp.509

[12] Okamoto K, Suzuki K, Takada T, Strasberg SM, Asbun HJ, Endo I, Iwashita Y, Hibi T, Pitt HA, Umezawa A, Asai K, Han HS, Hwang TL, Mori Y, Yoon YS, Huang WS, Belli G, Dervenis C, Yokoe M, Kiriyama S, Itoi T, Jagannath P, Garden OJ, Miura F, Nakamura M, Horiguchi A, Wakabayashi G, Cherqui D, de Santibanes E, Shikata S, Noguchi Y, Ukai T, Higuchi R, Wada K, Honda G, Supe AN, Yoshida M, Mayumi T, Gouma DJ, Deziel DJ, Liau KH, Chen MF, Shibao K, Liu KH, Su CH, Chan ACW, Yoon DS, Choi IS, Jonas E, Chen XP, Fan ST, Ker CG, Gimenez ME, Kitano S, Inomata M, Hirata K, Inui K, Sumiyama Y, Yamamoto M (2018) Tokyo guidelines 2018: flowchart for the management of acute cholecystitis. J Hepatobiliary Pancreat Sci 25(1):55–72 29045062 10.1002/jhbp.516

[13] Therapeutic Guidelines (2023) Therapeutic Guidelines Limited, Melbourne, https://www.tg.org.au. Cited 1 June 2023

[14] Australian Commission on Safety and Quality in Health Care (2021) AURA 2021 fourth Australian report on antimicrobial use and resistance in human health. ACSQHC, Sydney

[15] Clinical Excellence Commission (2021) Healthcare Associated Infection (HAI) clinical Indicator Manual. Clinical Excellence Commission, Sydney

[16] The Australian Council on Healthcare Standards (2022) ACHS 2022 clinical Indicator Program Information. NSW

[17] Friedman ND, Kaye KS, Stout JE, McGarry SA, Trivette SL, Briggs JP, Lamm W, Clark C, MacFarquhar J, Walton AL, Reller LB, Sexton DJ (2002) Health care–associated bloodstream infections in adults: a reason to change the accepted definition of community-acquired infections. Ann Intern Med 137(10):791–797 12435215 10.7326/0003-4819-137-10-200211190-00007

[18] Hall KK, Lyman JA (2006) Updated review of blood culture contamination. Clin Microbiol Rev 19(4):788–802 17041144 10.1128/CMR.00062-05 PMC1592696

[19] Gomi H, Solomkin JS, Schlossberg D, Okamoto K, Takada T, Strasberg SM, Ukai T, Endo I, Iwashita Y, Hibi T, Pitt HA, Matsunaga N, Takamori Y, Umezawa A, Asai K, Suzuki K, Han HS, Hwang TL, Mori Y, Yoon YS, Huang WS, Belli G, Dervenis C, Yokoe M, Kiriyama S, Itoi T, Jagannath P, Garden OJ, Miura F, de Santibañes E, Shikata S, Noguchi Y, Wada K, Honda G, Supe AN, Yoshida M, Mayumi T, Gouma DJ, Deziel DJ, Liau KH, Chen MF, Liu KH, Su CH, Chan ACW, Yoon DS, Choi IS, Jonas E, Chen XP, Fan ST, Ker CG, Giménez ME, Kitano S, Inomata M, Mukai S, Higuchi R, Hirata K, Inui K, Sumiyama Y, Yamamoto M (2018) Tokyo guidelines 2018: antimicrobial therapy for acute cholangitis and cholecystitis. J Hepatobiliary Pancreat Sci 25(1):3–16 29090866 10.1002/jhbp.518

[20] The European Committee on Antimicrobial Susceptibility Testing (2017) EUCAST guidelines for detection of resistance mechanisms and specific resistances of clinical and/or epidemiological importance. https://www.eucast.org/resistance_mechanisms. Cited 2 December 2023

[21] Australian Bureau of Statistics (2021) 2021 Census. Australian Bureau of Statistics, https://www.abs.gov.au/census. Cited 3 November 2023

[22] Lee CC, Chang IJ, Lai YC, Chen SY, Chen SC (2007) Epidemiology and prognostic determinants of patients with bacteremic cholecystitis or cholangitis. Am J Gastroenterol 102(3):563–569 17335448 10.1111/j.1572-0241.2007.01095.x

[23] Kruis T, Güse-Jaschuck S, Siegmund B, Adam T, Epple H-J (2020) Use of microbiological and patient data for choice of empirical antibiotic therapy in acute cholangitis. BMC Gastroenterol 20(1):65 32164573 10.1186/s12876-020-01201-6 PMC7066745

[24] Solomkin JS, Mazuski JE, Bradley JS, Rodvold KA, Goldstein EJC, Baron EJ, O’Neill PJ, Chow AW, Dellinger EP, Eachempati SR, Gorbach S, Hilfiker M, May AK, Nathens AB, Sawyer RG, Bartlett JG (2010) Diagnosis and management of complicated intra-abdominal infection in adults and children: guidelines by the Surgical Infection Society and the Infectious Diseases Society of America. Clin Infect Dis 50(2):133–164 20034345 10.1086/649554

[25] Traub WH, Leonhard B (1995) Piperacillin tazobactam compared with co-amoxiclav, ampicillin plus sulbactam and timentin against beta-lactamase-producing clinical isolates of Escherichia coli, Klebsiella pneumoniae and Klebsiella oxytoca. Chemotherapy 41(5):345–352 8521736 10.1159/000239366

[26] Ling W, Cadavid-Restrepo A, Furuya-Kanamori L, Harris PNA, Paterson DL (2022) Incidence and predictors of Escherichia coli producing extended-spectrum beta-lactamase (ESBL-Ec) in Queensland, Australia from 2010 to 2019: a population-based spatial analysis. Epidemiol Infect 150:e178 36285816 10.1017/S0950268822001637 PMC9987021

[27] Tamma PD, Aitken SL, Bonomo RA, Mathers AJ, van Duin D, Clancy CJ (2022) Infectious Diseases Society of America Guidance on the treatment of AmpC β-Lactamase-producing enterobacterales, Carbapenem-Resistant Acinetobacter baumannii, and Stenotrophomonas maltophilia infections. Clin Infect Dis 74(12):2089–2114 34864936 10.1093/cid/ciab1013

