# Supplementary material for: Epidemiology of biliary tract-associated bloodstream infections and adequacy of empiric therapy: an Australian population-based study
Source: Eur J Clin Microbiol Infect Dis. 2024 Jul 10;43(9):1753–60. doi: 10.1007/s10096-024-04894-9 (PMC11349862; doi:10.1007/s10096-024-04894-9)
Supplement: Supplementary file 1 — Supplementary Material 1 [file 10096_2024_4894_MOESM1_ESM.docx]

**Supplementary Table 1**. All organisms isolated among patients with biliary tract-associated bloodstream infection

| **Organism** | **Cholecystitis (n=1746)** | **Cholangitis**  **(n=2658)** |
| --- | --- | --- |
| *Escherichia coli* | 937 (53.7%) | 1,346 (50.6%) |
| *Klebsiella* species | 317 (18.2%) | 606 (22.8%) |
| Other Enterobacterales | 136 (7.8%) | 215 (8.1%) |
| Anaerobes | 99 (5.7%) | 52 (2.0%) |
| *Enterococcus* species | 79 (4.5%) | 214 (8.1%) |
| *Staphylococcus aureus* | 36 (2.1%) | 16 (0.6%) |
| *Streptococcus anginosus* group | 43 (2.5%) | 36 (1.4%) |
| Other Gram negatives | 38 (2.2%) | 82 (3.1%) |
| Other streptococci | 28 (1.6%) | 22 (0.8%) |
| *Pseudomonas* *species* | 18 (1.0%) | 64 (2.4%) |
| Other Gram positives | 14 (0.8%) | 5 (0.2%) |
| *Candida rugosa* | 1 (0.1%) | 0 |
